# Alpha-1-antitrypsin-deficiency is associated with lower cardiovascular risk: an approach based on federated learning

**DOI:** 10.1186/s12931-023-02607-y

**Published:** 2024-01-18

**Authors:** Daniela Zöller, Christian Haverkamp, Adeline Makoudjou, Ghislain Sofack, Saskia Kiefer, Denis Gebele, Michelle Pfaffenlehner, Martin Boeker, Harald Binder, Kapil Karki, Christian Seidemann, Bernd Schmeck, Timm Greulich, Harald Renz, Stefanie Schild, Susanne A. Seuchter, Dativa Tibyampansha, Roland Buhl, Gernot Rohde, Franziska C. Trudzinski, Robert Bals, Sabina Janciauskiene, Daiana Stolz, Sebastian Fähndrich

**Affiliations:** 1 Institute of Medical Biometry and Statistics, Faculty of Medicine and Medical Centre – University of Freiburg, Freiburg, Germany; 2 Freiburg Centre for Data Analysis and Modelling, University of Freiburg, Freiburg, Germany; 3 Institute of Digitalization in Medicine, Faculty of Medicine and Medical Centre – University of Freiburg, Freiburg, Germany; 4 Institute of Artificial Intelligence and Informatics in Medicine, Medical Centre Rechts der Isar, School of Medicine, Technical University of Munich, Munich, Germany; 5 Data Integration Centre, Medical Faculty, Philipps-University Marburg, Marburg, Germany; 6 Institute for Lung Research, Universities of Giessen and Marburg Lung Centre, Philipps-University Marburg, Marburg, Germany; 7 Department of Medicine, Pulmonary and Critical Care Medicine, University Hospital Giessen and Marburg, Philipps-University Marburg, Marburg, Germany; 8 German Centres for Lung Research (DZL) and for Infectious Disease Research (DZIF), SYNMIKRO Centre for Synthetic Microbiology, Philipps-University Marburg, Marburg, Germany; 9 Institute of Laboratory Medicine, German Centre for Lung Research (DZL) and the Universities of Giessen and Marburg Lung Centre (UGMLC), Philipps-University Marburg, Marburg, Germany; 10 Medical Centre for Information and Communication Technology, University Hospital Erlangen, Erlangen, Germany; 11 Institute of Medical Biostatistics, Epidemiology and Informatics, University Medical Centre of the Johannes Gutenberg-University Mainz, Mainz, Germany; 12 Pulmonary Department, University Medical Centre of the Johannes Gutenberg-University Mainz, Mainz, Germany; 13 Department of Respiratory Medicine, Medical Clinic I, Goethe University Frankfurt, University Hospital, Frankfurt/Main, Germany; 14 Department of Pneumology and Critical Care Medicine, German Centre for Lung Research (DZL), Translational Lung Research Centre Heidelberg (TLRC-H), University of Heidelberg, Thoraxklinik, Heidelberg, Germany; 15 Department of Internal Medicine V - Pulmonology, Allergology, Critical Care Medicine, Saarland University Medical Centre, Saarland University Hospital, 66421 Homburg/Saar, Germany; 16 Department of Pulmonary and Infectious Diseases and BREATH German Centre for Lung Research (DZL), Hannover Medical School, Hannover, Germany; 17 Department of Pneumology, University Medical Centre Freiburg, Freiburg, Germany

**Keywords:** COPD, Alpha-1-antitrypsin deficiency, Troponin, Cholesterol, Cardiovascular

## Abstract

**Background:**

Chronic obstructive pulmonary disease (COPD) is an inflammatory multisystemic disease caused by environmental exposures and/or genetic factors. Inherited alpha-1-antitrypsin deficiency (AATD) is one of the best recognized genetic factors increasing the risk for an early onset COPD with emphysema. The aim of this study was to gain a better understanding of the associations between comorbidities and specific biomarkers in COPD patients with and without AATD to enable future investigations aimed, for example, at identifying risk factors or improving care.

**Methods:**

We focused on cardiovascular comorbidities, blood high sensitivity troponin (hs-troponin) and lipid profiles in COPD patients with and without AATD. We used clinical data from six German University Medical Centres of the MIRACUM (Medical Informatics Initiative in Research and Medicine) consortium. The codes for the international classification of diseases (ICD) were used for COPD as a main diagnosis and for comorbidities and blood laboratory data were obtained. Data analyses were based on the DataSHIELD framework.

**Results:**

Out of 112,852 visits complete information was available for 43,057 COPD patients. According to our findings, 746 patients with AATD (1.73%) showed significantly lower total blood cholesterol levels and less cardiovascular comorbidities than non-AATD COPD patients. Moreover, after adjusting for the confounder factors, such as age, gender, and nicotine abuse, we confirmed that hs-troponin is a suitable predictor of overall mortality in COPD patients. The comorbidities associated with AATD in the current study differ from other studies, which may reflect geographic and population-based differences as well as the heterogeneous characteristics of AATD.

**Conclusion:**

The concept of MIRACUM is suitable for the analysis of a large healthcare database. This study provided evidence that COPD patients with AATD have a lower cardiovascular risk and revealed that hs-troponin is a predictor for hospital mortality in individuals with COPD.

**Supplementary Information:**

The online version contains supplementary material available at 10.1186/s12931-023-02607-y.

## Background

Chronic obstructive pulmonary disease (COPD) is an inflammatory and multifactorial disease [1], according to the World Health Organization data, it is the third leading cause of mortality worldwide [2]. The COPD phenotype results from the complex interplay of environmental, health and genetic risk factors [1]. A typical COPD patient often suffers from one or more comorbidities, specifically cardiovascular diseases (CVDs). There are many similarities between COPD and CVD and there are common risk factors, the most important of which is cigarette smoking. Persistent systemic inflammation, increased senescence, changes in the microbiome, and/or a genetic predisposition are determinants for coexistent comorbidities, such as CVDs but also osteoporosis, intellectual deterioration, muscle hypotrophy and cachexia [1, 3, 4]. Therefore, one of the goals of COPD research is to recognize disease phenotypes and to develop biomarkers that enable clinicians to identify individuals with increased risk for comorbidities and mortality, and those in need of a more intensive personalized treatment. Inherited Alpha-1-Antitrypsin-Deficiency (AATD) is a genetic predisposition to COPD and pulmonary emphysema that affects 0.01–0.02% of the population in Europe and that makes the lungs more vulnerable to inhaled harmful particles [5–7]. The most common AATD genotypes are Pi*Z (Glu342Lys) and Pi*S (Glu264Val) resulting from a point mutation in the SERPINA 1 gene encoding the alpha-1antitrypsin (AAT) protein, a broad-spectrum protease inhibitor and immunomodulatory protein [8–11]. The Pi*Z mutation in the SERPINA1 gene leads to AAT polymerization, intracellular accumulation, and low circulating levels of AAT [12–15].

As mentioned above, COPD patients have a high prevalence of cardiovascular risk factors and comorbidities [17]. However, knowledge about the prevalence of CVDs in patients with AATD-related emphysema remains limited and controversial. Greulich et al. [16] evaluated health insurance data, unadjusted for important confounders such as smoking status, age, gender and body mass index (BMI), and showed a significantly lower cardiovascular risk and comorbidity profile in COPD patients with AATD compared to those without AATD.

In a large prospective German multicentre cohort study (COSYCONET), the authors considered smoking, age, gender, and BMI as confounding factors of COPD and investigated comorbidities in stable COPD patients with and without AATD [17]. Here, in line with the previous study, the authors reported that patients with AATD-related COPD have a significantly lower prevalence for cardiovascular comorbidities. In contrast, a recent study from US insurance databases did not identify significant lower prevalence of cardiovascular comorbidities associated with AATD-related COPD [18].

Therefore, the aim of this study was to investigate in non-AATD and AATD COPD patients: (i) comorbidity profile (ii) high sensitivity troponin (hs-troponin) and (iii) blood glucose and lipid profiles. For this purpose, we analysed data in a real-life setting obtained from six medical centres of the Medical Informatics in Research and Care in University Medicine (MIRACUM) consortium [19].

## Methods

### Study population

We conducted a longitudinal study, which included patients diagnosed with COPD (primary or secondary diagnosis) during their hospitalization at one of the university hospital centres (Erlangen, Frankfurt, Freiburg, Giessen, Mainz and Marburg) pseudonymised as sites A to F. The data acquisition periods vary depending on the clinical centre, however, in each case the starting year has at least one AATD patient enrolled (for details see Supplementary). The university hospital centres belong to the MIRACUM consortium uniting ten university hospitals, two universities of applied sciences and one industrial partner spread over seven German states. The aim of the MIRACUM consortium is to make clinical data usable for research projects via modular, scalable and federated data integration centres [19–21]. COPD patients were diagnosed based on a diagnosis coded as J44 or according to the International Classification of Diseases revision 10 (ICD-10) [21]. In total, we were able to extract 112,852 visit^1^ information of 43,057 COPD patients with an average visit number of 2.19 per individual. Patients having AATD were defined based on a diagnosis code of E88.0 of the ICD-10 [22].

### Data extraction and handling

Routine data were collected within the participating hospitals’ routine care IT systems for all individuals under the local hospital laws. The data were retrieved from the database using the SQL language, pseudonymised and then imported into a standardized i2b2 database schema [23]. Additional data wrangling transformations were performed using the statistical software R [24]. In particular, continuous measurements that can be collected multiple times per visit were averaged across one visit to obtain one value per visit. Due to data protection / IT security constraints, an adapted DataSHIELD (Data Aggregation Through Anonymous Summary-statistics from Harmonised Individual levEL Databases) implementation was used for the analyses [25, 26], where the sensitive data remain within each hospital centre and only anonymous aggregated data are shared. Using specific statistical methods, one can still obtain the same analyses results with this framework as with locally pooled data for specific statistical models [27]. Each participating site uploaded selected cohort data from i2b2 database into its local DataSHIELD Open Policy Agent Layer (OPAL) server and access was granted to the analyst at each of the participating sites. This approach enabled the anonymous data analysis in line with the EU General Data Protection Regulation [28]. All six local ethics committees approved the study protocol.

### Measurements

For each visit of a COPD patient, the covariate information was extracted. Demographic characteristics included age at admission in years, gender and nicotine abuse indicated with an ICD-10 diagnosis F17. Considering that BMI is a key confounding factor to take into account in the analysis of AATD, an attempt was made to extract this data. However, the data had to be discarded subsequently due to an excessive number of missing values. All comorbidities were recorded as binary variables indicating the presence or absence of the comorbidity, and we assumed that a missing coded comorbidity indicated the absence of that comorbidity. Cardiovascular comorbidities included acute ischemic stroke, angina pectoris, arrhythmias, atrial fibrillation, carotid stenosis, coronary heart diseases, coronary sclerosis, heart failure, hypertension, ischemic heart, myocardial infarction, peripheral artery diseases and valvular diseases. Other diseases considered were bronchiectasis, diabetes, emphysema, liver diseases and renal failure. In addition, numerous laboratory parameters were retrieved. Laboratory biomarkers included triglycerides, Low Density Lipoprotein (LDL), High Density Lipoprotein (HDL), cholesterol, transaminases; Alanine Aminotransferase (ALAT or GPT) and Aspartate Aminotransferase (ASAT or GOT), glycosylated haemoglobin (HbA1c), platelets, haemoglobin and blood glucose. A high-sensitivity troponin was also included as a heart function biomarker. Neutrophils, lymphocytes, procalcitonin and C-reactive protein (CRP) were used as biomarkers related to systemic inflammation. Only complete cases were included in the analysis. A summary of the number of missing data per laboratory parameter can be found in Supplementary S5. Data extraction summaries based on ICD-10 codes for comorbidities and LOINC (Logical Observation Identifiers Names and Codes) codes for laboratory parameters are presented in Supplementary S2.

### Statistical analysis

The statistical analysis was performed within the DataSHIELD framework. Descriptive statistics were presented as means and standard deviations (SD). Frequencies and percentages were used to report distributions of categorical variables. The χ^2^-test was used to compare the prevalence of the different comorbidities between the two COPD groups (AATD versus non-AATD). The mean laboratory blood glucose and lipid profiles in both groups were compared using Student’s t-test. Generalized linear mixed effects regression models with a random effect per patient to account for multiple visits per patient and adjustments (age, gender and nicotine abuse) were used to estimate the effects of AATD for COPD patients on the odds of having a specific comorbidity. Similarly, in-hospital mortality (binary coded as discharged alive or not) associated with the biomarker hs-troponin was predicted in both groups. Specifically, we used a logistic regression model (family: binomial, link: logit). Analogously, linear mixed effects models including a random effect per patient and adjustments were used to estimate the impact of blood glucose and lipid biomarkers on comorbidity status (family: gaussian, link: identity). The corresponding forest plots were generated with the forester package in R [29]. The statistical analyses were performed using DataSHIELD version 6.1 [27] and R version 3.5.2 [24]. The reported probabilities values are two-sided and a threshold *p*-value of 0.05 was used to infer exploratory statistical significance.

## Results

### Patient characteristics

Table 1 shows the characteristics of all patients included in this study (see Supplementary S6 for site-specific details). In total, 43,057 COPD patients were enrolled, of whom 42,311 (98.27%) had no reported diagnosis for AATD and 746 (1.73%) had AATD. This COPD cohort included more males than females (61.67% vs. 38.33%), which was also true for non-AATD and AATD COPD sub-cohorts. Approximately 12% of all COPD patients were diagnosed as nicotine abuser compared to 8.6% of the AATD-related COPD patients. The average age at first hospital admission was 65.87 years for AATD-related, 70.17 years for non-AATD-related and 70.13 years for all COPD patients. The average number of hospital visits was higher for AATD patients than for Non-AATD patients (see Table 1). Only 3.22% of the COPD patients were coded as having asthma (see Strengths and limitations). The biomarkers, such as neutrophils, lymphocytes, procalcitonin and C-reactive protein (CRP), showed a higher average while the average value for haemoglobin and platelets was lower in AATD-related COPD patients. The higher CRP levels could hypothetically be related to the reduced anti-inflammatory effect of AAT.

**Table 1:** Baseline characteristics of the combined study population. For ’Number of individuals’ the percentage values correspond to the total number of COPD patients, for gender and nicotine abuse to the specific sub-cohort (i.e., COPD total, non-AATD, AATD, respectively). *p*-values are calculated using the Chi-squared test for categorical variables or t-test for comparing the mean values

Variable	COPD total	AATD	Non-AATD	*p*-value
Number of individuals	43,057	746 (1.73%)	42,311 (98.27%)	
Gender (Male)	26,554 (61.67%)	475 (63.67%)	26,079 (61.64%)	0.273
Nicotine Abuse^1^	6897 (16.02%)	170 (22.79%)	6727 (15.9%)	< 0.001
^∗^Age at first admission	70.13 (12.28)	65.87 (10.84)	70.17 (12.27)	< 0.001
^∗^Age at last discharge	70.47 (12.9)	66.51 (12.56)	70.5 (12.9)	< 0.001
^∗^Number of visits	2.19 (2.52)	3.08 (3.38)	2.18 (2.5)	< 0.001
Asthma^2^	830 (3.22%)	11 (1.82%)	819 (3.25%)	0.06412
^∗^Neutrophils^3^	8.53	8.95 (6.7)	8.52 (6.78)	0.4829
^∗^Haemoglobin^4^	50.87	23.8 (9.86)	51.31 (13.83)	< 0.001
^∗^Platelets	264.88	246.1 (128.3)	265.17 (120.76)	0.0001
^∗^Lymphocytes^3^	3.15	4.11 (7.67)	3.14 (7.07)	0.0688
^∗^Procalcitonin^5^	2.07	2.09 (7.85)	2.07 (11.1)	0.9738
^∗^CRP	42.94	60.19 (73.99)	42.65 (62.98)	< 0.001

^*^ Presented as mean (SD), AATD: Alpha 1-Antitrypsin Deficiency, ^1^Results are just for five sites due to disclosure risks in the Site C cohort, ^2^ Results are for all sites except for Site B und Site D, ^3^ differences between groups calculated for all sites except for Site E and Site F, ^4^ differences between groups calculated for all sites except for Site A, ^5^ differences between groups calculated for all sites except for Site E

In addition, we examined the influence of the inflammation biomarkers on the biomarker hs-troponin. Table 2 shows the results of the univariate linear regression for the enitre COPD cohort. The lack of a significant impact of neutrophils, procalcitonin, and CRP on hs-troponin led to exclusion of inflammation parameters from further analyses.

**Table 2:** Influence of Biomarkers related to systemic inflammation on hs-troponin using univariate linear regression

Variable	Estimate	Std. Error	Lower CI	Upper CI	*p*-value
Neutrophils	-0.05	0.20	-0.44	0.34	0.807
Haemoglobin	-0.20	0.17	-0.54	0.14	0.242
Platelets	0.01	0.003	0.001	0.01	0.026
Lymphocytes	-0.05	0.13	-0.31	0.20	0.671
Procalcitonin	-0.01	0.05	-0.11	0.10	0.901
CRP	-0.002	6.67E-03	-0.02	0.01	0.731

This model was fitted using pooled data from the Site A and Site E cohorts only

### Prevalence of comorbidities in AATD versus Non-AATD COPD patients

The overall comorbidities defined according to the ICD-10 codes were compared between AATD and Non-AATD patients (see Table 3). Our data revealed that AATD-related COPD patients have significantly lower cardiovascular risk profiles as compared to non-AATD COPD patients. Our results also show that hypertension is slightly less frequent among AATD compared to non-AATD patients (60.12% vs. 62.52%, *p*-value = 0.136). In addition, we observed a significantly lower prevalence of liver diseases (liver cirrhosis and liver fibrosis) and renal failure in AATD-related COPD as compared to non-AATD COPD patients (4.63% vs. 23.49% and 19.14% vs. 28.48%, respectively).

**Table 3:** Prevalence of comorbidities in total COPD cohort, and subgroups of non-AATD and AATD COPD patients. The data are given as a percentage. All *p*-values are calculated with the Chi-squared test for two groups

Comorbidity	COPD total	AATD	Non-AATD	*p*-value
Acute Ischemic Stroke	1.83	1.82	3.27	0.001
Angina Pectoris	3.82	3.83	2.84	0.143
Arrhythmias	28.31	28.15	46.31	< 0.001
Atrial Fibrillation	21.63	21.47	39.14	< 0.001
Bronchiectasis	99.48	99.49	98.77	0.004
Carotid Stenosis	2.43	2.43	2.14	0.651
Coronary Heart Disease	2.12	2.12	1.89	0.709
Coronary Sclerosis	26.54	26.51	29.20	0.067
Diabetes	26.58	26.52	32.79	< 0.001
Emphysema	98.48	98.51	95.38	< 0.001
Heart Failure	21.23	21.20	25.51	0.001
Hypertension	60.14	60.12	62.51	0.136
Ischemic Heart	30.61	30.59	33.51	0.056
Liver Disease	4.80	4.63	23.49	< 0.001
Myocardial Infarction	8.75	8.72	12.71	< 0.001
Peripheral vascular disorders	15.62	15.55	23.60	< 0.001
Renal Failure	19.22	19.14	28.48	< 0.001
Valvular Disease	9.50	9.42	18.44	< 0.001

### Lower cardiovascular disease risk in COPD patients with AATD

**Fig. 1:**
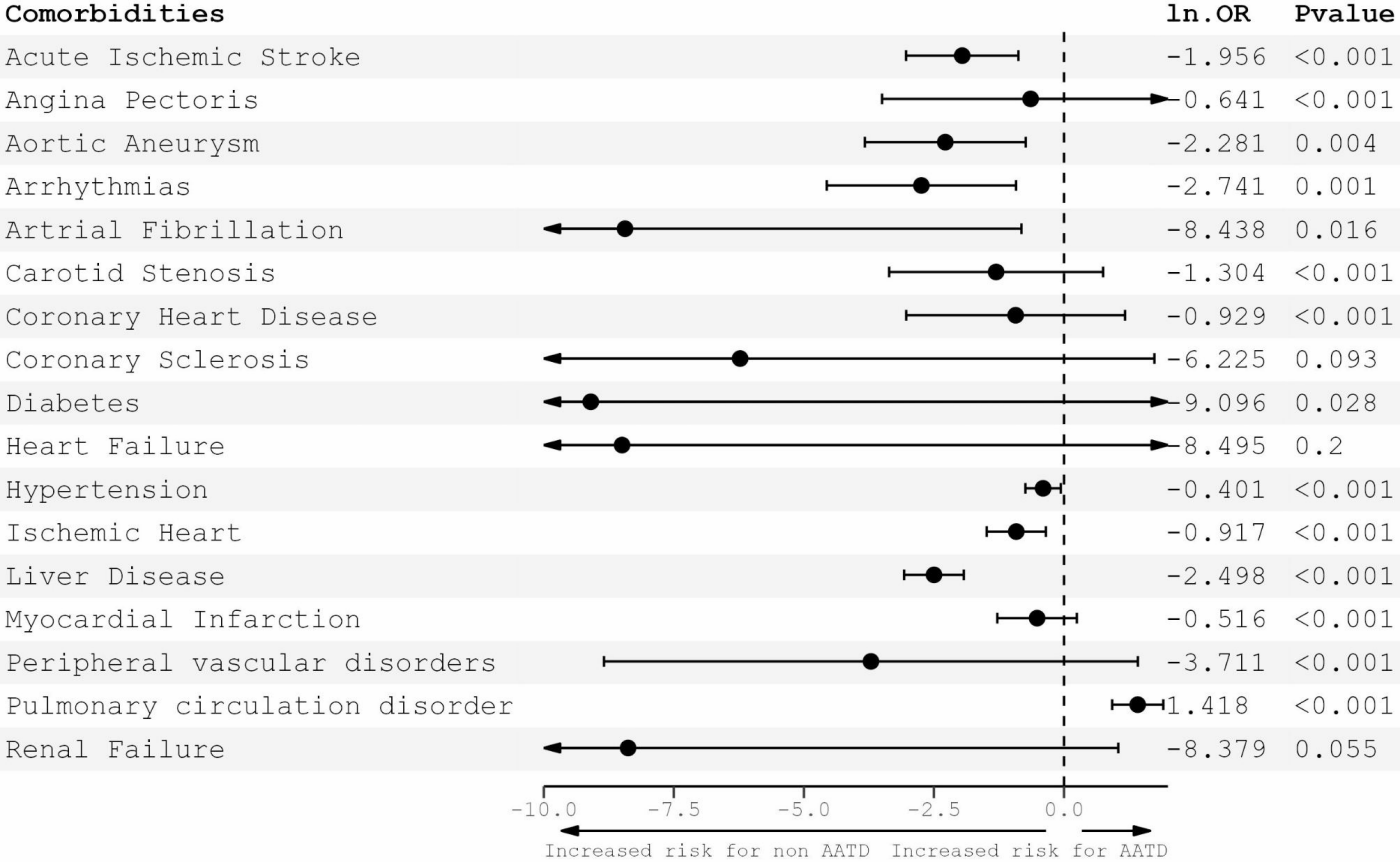
Forest plot showing the adjusted influence of AATD status on different comorbidities. Odd ratios (OR) are reported as ln (Odds), and confounders include age, gender and nicotine abuse

Next, we focused on the impact of AATD on the various comorbidities in COPD patients. As illustrated in Fig. 1, AATD patients show a reduced risk of developing cardiovascular diseases. In addition, our findings imply that AATD patients have a lower risk of developing other comorbidities such as diabetes mellitus (OR = -9.09, *p*-value = 0.028), liver diseases (OR = -2.49, *p*-value < 0.001) and renal failure (OR = -8.37, *p*-value = 0.055). On the contrary, they are more likely to develop pulmonary circulation disorders (OR = 1.42, *p*-value < 0.001).

### Reduced HbA1c and total cholesterol in COPD patients with AATD

Table 4a shows the unadjusted mean comparisons of different laboratory parameters between AATD and Non-AATD COPD patients. Our data show that when compared with Non-AATD, AATD COPD patients have lower plasma values of HbA1c (mean difference (MD) = -0.61, *p*-value = 0.001) and total cholesterol (MD = -7.72, *p*-value = 0.019). On the other hand, AATD patients present higher values of liver enzymes, specifically ALAT (MD = 20.16, *p*-value = 0.019) and ASAT (MD = 52.91, *p*-value = 0.016).

No significant differences were found for HDL (MD = -2.33, *p*-value = 0.275), LDL (MD = -4.28, *p*-value = 0.177), triglycerides (MD = -2.7, *p*-value = 0.016) and blood glucose (MD = 99.96, *p*-value = 0.844).

**Table 4:** Mean differences of laboratory parameters in all COPD patients. The confounders for the adjusted mean differences include age, gender and nicotine abuse. Unadjusted mean differences for AATD vs. Non-AATD COPD patients in laboratory parameters

Laboratory parameter	^+^Mean difference	Lower CI	Upper CI	*p*-value	Unit
**a**) Unadjusted mean differences for AATD vs. Non-AATD COPD patients in laboratory parameters					
ALAT	20.16	3.26	36.18	0.019	U/L
ASAT	52.91	9.74	95.91	0.016	U/L
Blood glucose	99.96	-818.6	1001.7	0.844	mg/dL
HbA1c	-0.61	-0.96	-0.35	0.001	mmol/mol
HDL	-2.33	-5.07	1.44	0.275	mg/dL
LDL	-4.28	-11.94	2.19	0.177	mg/dL
Triglyceride*	-2.7	-23.18	6.03	0.250	mg/dL
Total Cholesterol	-7.72	-17.55	-1.59	0.019	mg/dL
**b**) Adjusted mean differences for AATD vs. Non-AATD COPD patients in laboratory parameters					
ASAT	38.05	13.26	62.82	0.002	U/L
ALAT	11.77	1.49	22.05	0.024	U/L
Blood glucose	7.55	0.07	51,500,000	0.147	mg/dL
HbA1c	-0.52	-0.80	-0.23	0.001	mmol/mol
HDL	-2.24	-5.11	0.62	0.125	mg/dL
LDL	-5.03	-15.44	5.37	0.343	mg/dL
Triglyceride*	-10.57	n.a	n.a	0.205	mg/dL
Total Cholesterol	-11.04	-19.19	-2.88	0.007	mg/dL

^+^ The mean difference for AATD (n = 55) is relative to Non-AATD (n = 4365) and the *p*-value is that of the student’s t-test comparison. CI: Confidence Interval, U/L: Units per litre, mg/dl: Milligrams per decilitre, mmol/mol: Millimoles per mole, AATD: Alpha 1-Antitrypsin Deficiency, ASAT: Aspartate Aminotransferase, ALAT: Alanine Aminotransferase, HbA1c: Glycosylated haemoglobin, HDL: High Density Lipoprotein, LDL: Low Density Lipoprotein

^∗^The difference here includes all sites except Site F cohort (n.a is due to DataSHIELD disclosure controls)

The results of the generalized linear mixed effect models adjusted for gender, age and nicotine abuse (Table 4b) confirmed that plasma HbA1c (MD = -0.52, *p*-value = 0.001) as well as total cholesterol (MD = -11.04, *p*-value = 0.007) were significantly lower in AATD than in non-AATD COPD patients. The values of these laboratory parameters are consistent with the observed lower risk of cardiovascular comorbidities in AATD compared to non-AATD COPD.

### Predicting hospital mortality

We used hs-troponin levels as a biomarker to predict hospital mortality in the complete COPD cohort, and sub-cohorts of AATD and Non-AATD COPD patients. As shown in Table 5, hs-troponin was a poor predictor of hospital mortality in AATD patients (OR = 9.93, *p*-value = 0.330) while it was significant in predicting hospital mortality for Non-AATD and total cohort of COPD patients (OR = 1.85, *p*-value < 0.001).

**Table 5:** Table assessing the influence of hs-troponin on predicting hospital mortality

	Estimate	OR	lower.CI	upper.CI	p- value
**AATD (n = 68)**					
Gender (Ref: Male)	-0.38	0.68	-1.63	0.87	0.552
Age	0.06	1.07	0.01	0.12	0.029
Nicotine abuse (Ref: Non-smoker)	1.39	4.04	-0.62	3,41	0,174
Hs-troponin	2.29	9.93	-2.33	6.92	0.330
**Non AATD (n = 3465)**					
Gender (Ref: Male)	0.05	1.05	-0.09	0.18	0.489
Age	0.01	1.02	0.01	0,02	*<* 0.001
Nicotine abuse (Ref: Non-smoker)	-0.13	0.88	-0.31	0,05	0.157
Hs-troponin	0.62	1.85	0.26	0.97	*<* 0.001
**All COPD (n = 3533)**					
Gender (Ref: Male)	0.04	1.04	-0.09	0.17	0.525
Age	0.02	1.02	0.01	0.02	*<* 0.001
Nicotine abuse (Ref: Non-smoker)	-0.11	0.9	-0.29	0.07	0.220
AATD(Ref: Non-AATD)	0.82	2.26	0.17	1.46	0.012
Hs-troponin	0.62	1.85	0.26	0.97	*<* 0.001
AATD: hs-troponin	1.80	6.07	-2.42	6.03	0.403

This model was fitted using pooled data from the Site A and Site E cohorts only. AATD: Alpha 1-antitrypsin deficiency, hs-troponin: high sensitivity troponin

### Correlation of liver biomarkers in individuals with liver diseases

We also investigated to what extent liver biomarkers correlate with liver diseases in the entire COPD cohort and the sub-cohorts of AATD and non-AATD COPD patients. Liver diseases were defined as those covered by the coding algorithms for the Elixhauser comorbidities generated within the comorbidity package in R [30], including liver cirrhosis, liver fibrosis, liver failure, hepatitis, gastric and oesophageal varices. As presented in Table 6, COPD patients – overall and for the sub-cohorts AATD and non-AATD – without liver diseases show significantly lower values for ASAT and ALAT as compared to patients with liver diseases. In contrast, COPD patients without liver disease have significantly higher values for LDL (MD = 9.79, *p*-value < 0.001) and total cholesterol (MD = 16.04, *p*-value < 0.001). This applies to the sub-cohort of AATD COPD patients as well (LDL: MD = 9.86, *p*-value < 0.001; total cholesterol: MD = 16.08, *p*-value < 0.001).

**Table 6:** Adjusted mean effects of liver diseases on liver transaminases, LDL cholesterol and total cholesterol. Confounding factors include age, gender and nicotine abuse

Biomarker	*Mean Difference	Lower CI	Upper CI	*p*-value
**AATD (n = 170)**				
ALAT	-76.07	-105.49	-46.64	*<* 0.001
ASAT	-164.55	-259.93	-69.17	*<* 0.001
LDL	9.86	7.08	12.65	*<* 0.001
Total cholesterol	16.08	9.15	23.01	*<* 0.001
**Non-AATD (n = 3228)**				
ALAT	-35.34	-54.94	-15.73	*<* 0.001
ASAT	-96.47	-174.85	-18.09	0.016
LDL	31.01	-5.62	67.63	0.097
Total cholesterol	34.76	-18.98	88.51	0.205
**COPD (n = 3398)**				
ALAT	-85.12	-114.84	-55.55	< 0.001
ASAT	-192.88	-289.86	-95.91	*<* 0.001
LDL	9.79	5.70	13.88	*<* 0.001
Total cholesterol	16.04	8.38	23.70	*<* 0.001

* In this table, the difference is comparing transaminases between individuals without liver disease and those with liver disease. ASAT: Aspartate Aminotransferase, ALAT: Alanine Aminotransferase, LDL: Low Density Lipoprotein

## Discussion

This is the first study on AATD related COPD integrating data from several university hospitals in a distributed analysis approach utilizing the technical infrastructure of the MIRACUM data integration centres. Moreover, the MIRACUM database allowed the reassessment of plasma cardiac hs-troponin, as a major indicator of cardiovascular events and an independent predictor for mortality in patients with COPD [31].

First, we confirmed the already known relationship between AATD and an increased prevalence of bronchiectasis [17, 32]. On the other hand, our data revealed that AATD-related COPD patients have a much lower risk of a cardiovascular disease than non-AATD COPD patients. Accordingly, the prevalence of acute ischemic stroke, angina pectoris, arrhythmias, atrial fibrillation, carotid stenosis, coronary heart diseases, ischemic heart, liver diseases and cardiac infarction were significantly lower than in individuals with non-AATD COPD.

Our findings are also in line with data reported by Greulich et al. The authors also found lower cardiovascular comorbidity profiles in COPD patients with AATD by examining health insurance data, however, without accounting for the confounding factors like age, nicotine abuse and BMI [16]. The German prospective multicentre study COSYCONET also found a significantly lower prevalence of cardiovascular disease in AATD-related COPD patients [17]. Although the COSYCONET results were based on the adjustment of age, gender, BMI and pack years of cigarettes, patient selection bias cannot be totally excluded because significantly younger AATD patients were enrolled due to family screening. Unlike in the COSYCONET cohort study, we only considered patients having COPD as a primary or secondary diagnosis. Despite the above differences in study designs, previously published [16, 17] and current data support the notion that AATD-related COPD patients have a lower risk for cardiovascular diseases than Non-AATD COPD patients. The question remains why? According to the COSYCONET study, lower cardiovascular comorbidities in AATD than in non-AATD COPD patients can be associated with significantly lower plasma triglyceride concentrations and lower HbA1c in COPD patients with AATD [17]. In contrast to the results of COSYCONET, we could not confirm that the long-term blood sugar parameter HbA1c is reduced in AATD COPD [17]. A possible explanation for these conflicting findings could be that our current study is based on the larger patient cohort, which includes older individuals with AATD than in COSYCONET. On the other hand, our findings are in line with Bhattacharjee et al. who did not find a lower HbA1c in AATD patients with hepatic insufficiency [33].

Recently, Hamesch et al. [34] showed that Pi*ZZ AATD individuals have lower serum concentrations of LDL, VLDL and triglyceride, and related this finding to hepatic damage caused by intracellular accumulation of misfolded Z-AAT protein. These authors also provided evidence that Pi*Z-overexpressing mice have reduced expression of genes involved in lipid secretion and develop liver steatosis [34].

Our results revealed that plasma levels of total cholesterol are lower in patients with AATD, but not in terms of LDL cholesterol, HDL cholesterol and triglycerides (routinely measured serum total cholesterol is a sum of lipoprotein particles with high, low, intermediate and very low density (HDL, LDL, IDL a VLDL). Out of these, VLDL particles are almost exclusively synthesized in the liver and secreted into the bloodstream. IDL and LDL particles are derived from VLDL after the loss of free fatty acids. HDL particles are also largely produced in the liver, but their production in the small intestine is also important [35].

Because liver diseases lead to a decrease in serum levels of cholesterol and triglycerides [36], this might explain why individuals with AATD - which is often associated with liver disease [37] - have lower levels of total cholesterol. In fact, lower cholesterol levels are in turn associated with a lower risk of cardiovascular diseases [38]. Patients with AATD have a more vulnerable liver, indicated by the elevated liver transaminases ASAT and ALAT, which were significantly higher in patients with AATD in this study. This is congruent with the results of COSYCONET that confirmed lower levels of total cholesterol and a lower prevalence of cardiovascular comorbidities in individuals with AATD [17]. Patients with AATD might secrete less total-cholesterol due to the accumulation of misfolded AAT in the hepatocytes and thus might have a lower prevalence of cardiovascular comorbidities.

Contrary to expectations, the real life data showed fewer liver diseases in patients with AATD than in COPD patients without AATD. The lower prevalence of liver disease in AATD is most likely due to bias, since it is undisputed that individuals with AATD show a higher association with liver disease [37]. A very likely reason could be that the liver diseases were not always recorded in the hospital bills, especially since only diagnoses that lead to increased reimbursement by the health insurance companies are coded. This also shows that the coded data should always be interpreted from the point of view of coding practice, which is a limitation of such big data studies. Another reason could also be the lack of awareness in clinical routine that AATD can also be a disease of the liver [39]. For this reason, available laboratory values should also be included in the interpretation, such as the elevated transaminases ALAT and ASAT in AATD as done in our study.

Moreover, the evaluation of ‘big data’ in MIRACUM is also suitable for the analysis of the biomarker hs-troponin in terms of prediction of mortality in individuals with COPD. We confirmed the results of Waschki et al. from the German prospective cohort study COSYCONET, that hs-troponin is a suitable biomarker for estimating mortality in subjects with stable COPD. While Waschki et al. examined subjects with stable COPD, we were able to analyse real life data that also includes patients with exacerbated COPD.

### Strengths and limitations

One strength of our study is the consideration of a big sample size from the MIRACUM consortium data integration centres to provide routinely collected data on Non-AATD and AATD COPD. The use of the DataSHIELD platform enabled the analysis of data from different cohorts of the MIRACUM consortium, hence increasing the statistical power of our analysis while preserving the privacy of patient data. Another advantage of MIRACUM was that, in contrast to COSYCONET, we had no selection bias in AAT patients. A further advantage was that important confounders for comorbidities could be taken into account. Some variables and biomarkers (e.g., hs-troponin) were not recorded across all participating cohorts with the consequence, that some results such as prevalence, effect estimates and predictions could not be generalized to our entire study population but rather to individual cohorts. In addition, despite the use of pooled data, the proportion of AATD patients was still relatively small. This is attributed to the rarity of this orphan disease and might have affected the significance as well as the precision of some differences. Another limitation is that BMI was only rarely recorded and therefore not available for the analysis. Furthermore, we were only able to adjust for a coded nicotine abuse and not the current smoking status. In addition, we could not provide any information on AAT augmentation therapy of patients with AATD, which could also have an impact on blood lipids, since this is not coded separately in Germany [40]. Up to now, the data available in the MIRACUM consortium are mostly coded with reimbursement in mind, and not research. Thus, so-called upcoding (i.e., using fitting ICD-10 and OPS codes with the highest reimbursement amount, not the highest clinical relevance) and selective coding (i.e., information not relevant for reimbursement is only coded rarely) can lead to biased results. Data on exacerbations were not available because they were not always coded. Currently, we were unable to consider lung function information because these measurements were too heterogeneous from site to site and were often missing. Nevertheless, the combination of the data from six university hospitals leads to a reliable statement due to the large number of cases.

## Conclusion

We conclude that the concept of MIRACUM is feasible for the analysis of a large healthcare database that provided important data, especially for orphan diseases. We have proved evidence that COPD patients with AATD have a lower cardiovascular risk. In addition, our real world data showed that hs-troponin is a predictor for hospital mortality in patients with COPD.

### Electronic supplementary material

Below is the link to the electronic supplementary material.

Supplementary Material 1: Figure 1. Flow chart demonstrating the number of AATD and Non-AATD patients at each research database and the respective recording period

Supplementary Material 2: ICD and LOINC codes for comorbidities

Supplementary Material 3: Site-specific prevalence of cardiovascular comorbidities tables

Supplementary Material 4: Site-specific forest plot showing the adjusted influence of AATD status on different comorbidities

Supplementary Material 5: Table 1: Summary of the variables used in the analysis including the amount of data missing for each laboratory values

Supplementary Material 6: Site-specific patient characteristics

## Data Availability

The access to the datasets generated and/or analysed during the current study are not publicly available but are available from the corresponding author on reasonable request.

## List of Abbreviations

AAT: Alpha-1antitrypsin
AATD: Alpha-1-antitrypsin deficiency
ALAT: Alanine Aminotransferase
ASAT: Aspartate Aminotransferase
CI: Confidence interval
COPD: Chronic obstructive pulmonary disease
CRP: C-reactive protein
CVD: Cardiovascular disease
DataSHIELD: Data Aggregation Through Anonymous Summary-statistics from Harmonised Individual levEL Databases
DRG: Diagnostic-Related Groups
HbA1c: Glycosylated haemoglobin
HDL: High Density Lipoprotein
hs-troponin: High sensitivity troponin
ICD-10: International Classification of Diseases revision 10
ICD: International Classification of Diseases
LDL: Low Density Lipoprotein
LOINC: Logical Observation Identifiers Names and Codes

## Footnotes

1 A visit corresponds to a case according to diagnostic-related groups (DRG).
