# Supplementary material for: Alpha-1-antitrypsin-deficiency is associated with lower cardiovascular risk: an approach based on federated learning
Source: Respir Res. 2024 Jan 18;25:38. doi: 10.1186/s12931-023-02607-y (PMC10797985; doi:10.1186/s12931-023-02607-y)
Supplement: Supplementary file 1 — Supplementary Material 1: Figure 1. Flow chart demonstrating the number of AATD and Non-AATD patients at each research database and the respective recording period [file 12931_2023_2607_MOESM1_ESM.docx]

## Supplementary S1

Flow chart demonstrating the number of AATD and Non-AATD patients at each research database


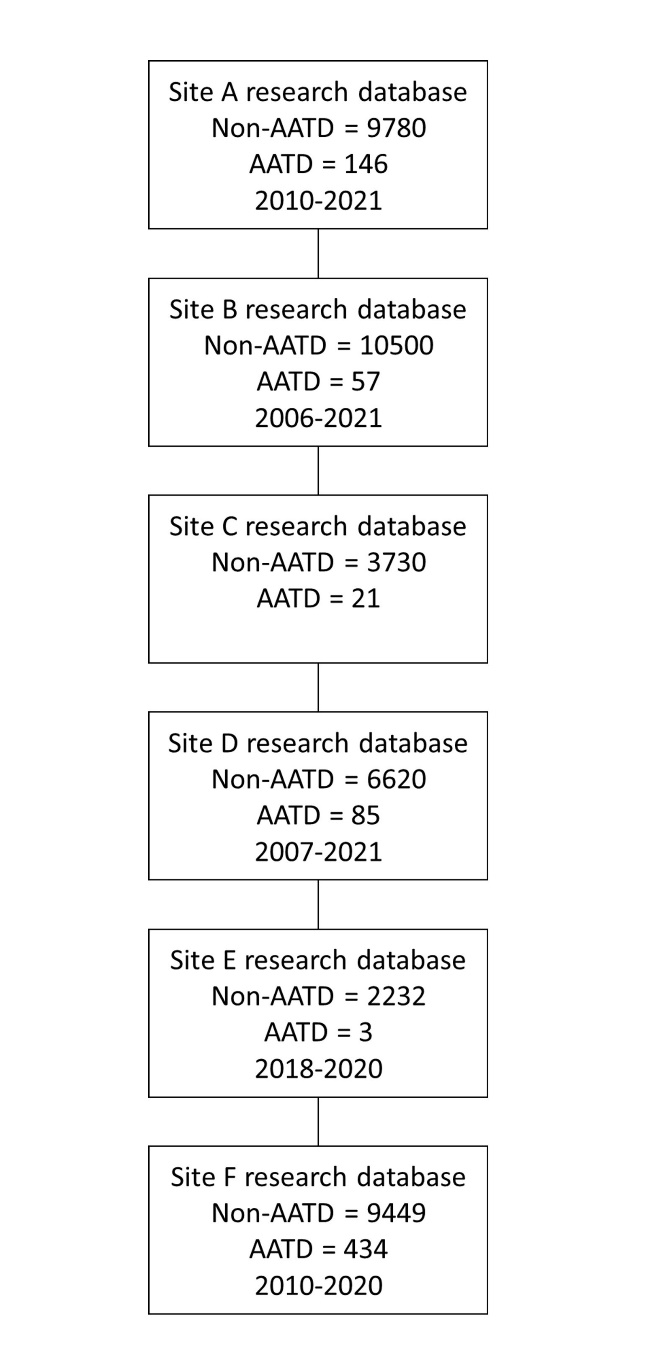


**Figure 1.** Flow chart demonstrating the number of AATD and Non-AATD patients at each research database and the respective recording period
