# Supplementary material for: Alpha-1-antitrypsin-deficiency is associated with lower cardiovascular risk: an approach based on federated learning
Source: Respir Res. 2024 Jan 18;25:38. doi: 10.1186/s12931-023-02607-y (PMC10797985; doi:10.1186/s12931-023-02607-y)
Supplement: Supplementary file 2 — Supplementary Material 2: ICD and LOINC codes for comorbidities [file 12931_2023_2607_MOESM2_ESM.docx]

**Table 1. List of all ICD codes used in the data extraction process**

| **Comorbidities** | **ICD-10 code used** |
| --- | --- |
| Myocardial infarction | I21, I25.2, I22 |
| Artrial fibrillation | I48 |
| AATD | E88.0 |
| COPD | J44 |
| Emphysemia | J43 |
| Bronchiectasis | J47 |
| Acute ischemic stroke | I63.x |
| Ischemic heart | I20-I25 |
| Pneumococcus | J13 |
| Pseudomonas | J15.1 |
| Coronary heart disease | I25.10 |
| Aortic aneurysm | I71.1 |
| Angina pectoris | I20 |
| Coronary sclerosis | I25.1 |
| Carotid stenosis | I66.x, I65.x |
| Peripheral vascular disorders | I70.x, I71.x, I73.1, I73.8, I73.9, I77.1, I79.0, I79.2, K55.1, K55.8, K55.9, Z95.8, Z95.9 |
| Hypertension | I10.x, I11.x-I13.x, I15.x |
| Arrhythmias | I44.1-I44.3, I45.6, I45.9, I47.x-I49.x, ROO.O, ROO.1, ROO.8, T82.1, Z45.0, Z95.0 |
| Diabetes | E10.0, E10.1, E10.9, E11.0, E11.1, E11.9, E12.0, E12.1, E12.9, E13.0, E13.1, E13.9, E14.0, E14.1, E14.9, E10.2-E10.8,  E11.2-E11.8, E12.2-E12.8, E13.2-E13.8, E14.2-E14.8 |
| Heart failure | I09.9, I11.0, I13.0, I13.2, I25.5, I42.0, 142.5-I42.9, I43.x, I50.x, P29.0 |
| Liver disease | B18.x, I85.x, I86.4, I98.2, K70.x, K71.1, K71.3-K71.5, K71.7, K72.x-K74.x, K76.0, K76.2-K76.9. Z94.4 |
| Chronic pulmonary disease | I27.8, 127.9, J40.x-J47.x, J60.x-J67.x, J68.4, J70.1, J70.3 |
| Pulmonary circulation disorders | I26.x, I27.x, I28.0, I28.8, I28.9 |
| Valvular disease | A52.0, I05.x-I08.x, I09.1, I09.8, I34.x-I39.x, Q23.O-Q23.3, Z95.2, Z95.4 |
| Renal failure | I12.0, I13.1, N18.x, NI9.x, N25.0, Z49.0-Z49.2, Z94.0, Z99.2 |

**Table 2. List of all LOINC codes used in the data extraction process**

| **Labor Parameter** | **LOINC Code used** |
| --- | --- |
| ALAT | 1742-6, 1743-4 |
| ASAT | 1920-8, 30239-8 |
| Blood glucose | 2339-0, 2345-7, 41653-7,30934-4 |
| HbA1c | 17856-6, 4548-4 |
| HDL | 2085-9, 49130-8 |
| HS-Troponin | 6598-7 |
| LDL | 13457-7, 49132-4, 2089-1 |
| ProBNP | 33762-6, 83107-3 |
| Triglycerides | 2571-8 |
| Total cholesterol | 2093-3 |
