# Supplementary material for: Alpha-1-antitrypsin-deficiency is associated with lower cardiovascular risk: an approach based on federated learning
Source: Respir Res. 2024 Jan 18;25:38. doi: 10.1186/s12931-023-02607-y (PMC10797985; doi:10.1186/s12931-023-02607-y)
Supplement: Supplementary file 3 — Supplementary Material 3: Site-specific prevalence of cardiovascular comorbidities tables [file 12931_2023_2607_MOESM3_ESM.docx]

**Prevalence and comorbidities**

| **Site A** | | | | |
| --- | --- | --- | --- | --- |
| **Comorbidity** | **AATD** | **Non-AATD** | **ALL** | **p-value** |
| Acute Ischemic Stroke | 2.32 | 6.10 | 2.36 | < 0.001 |
| Angina Pectoris | 1.57 | 0.00 | 1.56 | 0.085 |
| Arrhythmias | 25.51 | 28.05 | 25.54 | 0.404 |
| Artrial Fibrillation | 19.12 | 19.51 | 19.12 | 0.94 |
| Bronchiectasis | 98.79 | 95.12 | 98.76 | < 0.001 |
| Carotid Stenosis | 2.40 | 1.22 | 2.38 | 0.321 |
| Coronary Heart Disease | 1.65 | 3.25 | 1.67 | 0.09 |
| Coronary Sclerosis | 22.52 | 16.26 | 22.46 | 0.023 |
| Diabetes | 22.72 | 20.33 | 22.70 | 0.414 |
| Emphysemia | 95.86 | 86.99 | 95.78 | < 0.001 |
| Heart Failure | 13.43 | 13.82 | 13.43 | 0.931 |
| Hypertension | 53.16 | 39.43 | 53.03 | < 0.001 |
| Ischemic Heart | 25.61 | 19.92 | 25.55 | 0.05 |
| Liver Disease | 6.01 | 26.02 | 6.20 | < 0.001 |
| Myocardial Infarction | 7.43 | 5.69 | 7.41 | 0.362 |
| Peripheral vascular disorders | 17.85 | 15.04 | 17.82 | 0.288 |
| Renal Failure | 17.12 | 28.86 | 17.23 | < 0.001 |
| Valvular Disease | 6.75 | 11.79 | 6.80 | 0.003 |

| **Site B** | | | | |
| --- | --- | --- | --- | --- |
| **Comorbidity** | **AATD** | **Non-AATD** | **ALL** | **p-value** |
| Acute Ischemic Stroke | 1.59 | 4.62 | 1.59 | 0.146 |
| Angina Pectoris |  |  | 2.65 |  |
| Arrhythmias | 27.51 | 36.92 | 27.54 | 0.119 |
| Artrial Fibrillation | 20.78 | 29.23 | 20.80 | 0.128 |
| Bronchiectasis | 99.68 | 100.00 | 99.68 | > 0.05 |
| Carotid Stenosis |  |  | 2.18 |  |
| Coronary Heart Disease | 1.32 | 0.00 | 1.31 | 0.7 |
| Coronary Sclerosis | 26.45 | 15.38 | 26.42 | 0.06 |
| Diabetes | 26.67 | 23.08 | 26.66 | 0.607 |
| Emphysemia | 99.96 | 100.00 | 99.96 | > 0.05 |
| Heart Failure | 21.87 | 27.69 | 21.89 | 0.326 |
| Hypertension | 57.16 | 49.23 | 57.14 | 0.244 |
| Ischemic Heart | 30.20 | 21.54 | 30.17 | 0.166 |
| Liver Disease | 4.09 | 23.08 | 4.14 | < 0.001 |
| Myocardial Infarction | 6.88 | 9.23 | 6.89 | 0.616 |
| Peripheral vascular disorders | 14.48 | 18.46 | 14.49 | 0.463 |
| Renal Failure | 21.73 | 23.08 | 21.74 | 0.911 |
| Valvular Disease | 8.44 | 18.46 | 8.46 | 0.007 |

| **Site C** | | | | |
| --- | --- | --- | --- | --- |
| **Comorbidity** | **AATD** | **Non-AATD** | **ALL** | **p-value** |
| Acute Ischemic Stroke |  |  | 1.41 |  |
| Angina Pectoris | 4.49 | 0.00 | 4.47 | 0.509 |
| Arrhythmias | 29.20 | 33.33 | 29.21 | 0.795 |
| Artrial Fibrillation | 22.84 | 33.33 | 22.88 | 0.286 |
| Bronchiectasis | 99.61 | 100.00 | 99.61 | > 0.05 |
| Carotid Stenosis |  |  | 1.72 |  |
| Coronary Heart Disease |  |  | 2.97 |  |
| Coronary Sclerosis |  |  | 29.01 |  |
| Diabetes | 29.20 | 33.33 | 29.21 | 0.795 |
| Emphysemia |  |  | 99.45 |  |
| Heart Failure | 22.92 | 18.52 | 22.90 | 0.754 |
| Hypertension | 63.85 | 55.56 | 63.82 | 0.488 |
| Ischemic Heart |  |  | 31.74 |  |
| Liver Disease | 3.50 | 22.22 | 3.57 | < 0.001 |
| Myocardial Infarction |  |  | 8.96 |  |
| Peripheral vascular disorders | 16.80 | 37.04 | 16.88 | 0.011 |
| Renal Failure | 19.39 | 18.52 | 19.39 | > 0.05 |
| Valvular Disease | 13.41 | 22.22 | 13.44 | 0.291 |

###

| **Site D** | | | | |
| --- | --- | --- | --- | --- |
| **Comorbidity** | **AATD** | **Non-AATD** | **ALL** | **p-value** |
| Acute Ischemic Stroke | 1.77 | 3.70 | 1.78 | 0.251 |
| Angina Pectoris | 5.57 | 12.04 | 5.60 | 0.007 |
| Arrhythmias | 28.04 | 42.59 | 28.10 | 0.001 |
| Artrial Fibrillation | 21.83 | 37.04 | 21.90 | < 0.001 |
| Bronchiectasis | 99.81 | 100.00 | 99.81 | > 0.05 |
| Carotid Stenosis | 3.07 | 4.63 | 3.07 | 0.51 |
| Coronary Heart Disease | 3.97 | 5.56 | 3.98 | 0.553 |
| Coronary Sclerosis | 25.58 | 37.96 | 25.63 | 0.005 |
| Diabetes | 26.85 | 36.11 | 26.89 | 0.04 |
| Emphysemia | 98.79 | 97.22 | 98.78 | 0.298 |
| Heart Failure | 28.13 | 45.37 | 28.20 | < 0.001 |
| Hypertension | 59.82 | 67.59 | 59.86 | 0.122 |
| Ischemic Heart | 30.85 | 39.81 | 30.88 | 0.056 |
| Liver Disease | 4.99 | 18.52 | 5.04 | < 0.001 |
| Myocardial Infarction | 8.20 | 14.81 | 8.23 | 0.02 |
| Peripheral vascular disorders | 13.65 | 31.48 | 13.72 | < 0.001 |
| Renal Failure | 21.63 | 25.00 | 21.65 | 0.465 |
| Valvular Disease | 10.46 | 23.15 | 10.51 | < 0.001 |

| **Site E** | | | | |
| --- | --- | --- | --- | --- |
| **Comorbidity** | **AATD** | **Non-AATD** | **ALL** | **p-value** |
| Acute Ischemic Stroke |  |  | 2.19 |  |
| Angina Pectoris | 1.24 | 0 | 1.24 | > 0.05 |
| Arrhythmias |  |  | 28.64 |  |
| Artrial Fibrillation |  |  | 22.16 |  |
| Bronchiectasis | 99.85 | 100 | 99.85 | > 0.05 |
| Carotid Stenosis | 1.24 | 0 | 1.24 | > 0.05 |
| Coronary Heart Disease | 0.95 | 0 | 0.95 | > 0.05 |
| Coronary Sclerosis | 22.51 | 0 | 22.49 | 0.809 |
| Diabetes | 26.44 | 100 | 26.51 | 0.026 |
| Emphysemia | 99.70 | 100 | 99.70 | > 0.05 |
| Heart Failure |  |  | 20.12 |  |
| Hypertension | 64.29 | 100 | 64.32 | 0.492 |
| Ischemic Heart | 24.02 | 0 | 23.99 | 0.766 |
| Liver Disease | 3.70 | 0 | 3.70 | > 0.05 |
| Myocardial Infarction | 2.13 | 0 | 2.13 | > 0.05 |
| Peripheral vascular disorders | 14.18 | 0 | 14.17 | > 0.05 |
| Renal Failure |  |  | 16.21 |  |
| Valvular Disease |  |  | 7.51 |  |

| **Site F** | | | | |
| --- | --- | --- | --- | --- |
| **Comorbidity** | **AATD** | **Non-AATD** | **ALL** | **p-value** |
| Acute Ischemic Stroke | 1.57 | 1.70 | 1.57 | 0.956 |
| Angina Pectoris | 4.46 | 2.45 | 4.42 | 0.034 |
| Arrhythmias | 31.43 | 57.36 | 32.00 | < 0.001 |
| Artrial Fibrillation | 23.88 | 50.19 | 24.45 | < 0.001 |
| Bronchiectasis | 99.57 | 100.00 | 99.58 | 0.245 |
| Carotid Stenosis | 1.94 | 2.08 | 1.94 | 0.946 |
| Coronary Heart Disease | 1.58 | 0.75 | 1.56 | 0.183 |
| Coronary Sclerosis | 32.44 | 35.28 | 32.50 | 0.182 |
| Diabetes | 29.21 | 38.68 | 29.41 | < 0.001 |
| Emphysemia | 99.28 | 98.30 | 99.26 | 0.019 |
| Heart Failure | 20.45 | 26.98 | 20.59 | < 0.001 |
| Hypertension | 69.28 | 73.96 | 69.38 | 0.024 |
| Ischemic Heart | 36.94 | 40.19 | 37.01 | 0.137 |
| Liver Disease | 3.85 | 23.58 | 4.29 | < 0.001 |
| Myocardial Infarction | 13.58 | 16.04 | 13.63 | 0.117 |
| Peripheral vascular disorders | 16.21 | 26.04 | 16.42 | < 0.001 |
| Renal Failure | 15.62 | 30.19 | 15.94 | < 0.001 |
| Valvular Disease | 10.90 | 20.38 | 11.11 | < 0.001 |
