# Supplementary material for: Alpha-1-antitrypsin-deficiency is associated with lower cardiovascular risk: an approach based on federated learning
Source: Respir Res. 2024 Jan 18;25:38. doi: 10.1186/s12931-023-02607-y (PMC10797985; doi:10.1186/s12931-023-02607-y)
Supplement: Supplementary file 4 — Supplementary Material 4: Site-specific forest plot showing the adjusted influence of AATD status on different comorbidities [file 12931_2023_2607_MOESM4_ESM.docx]

Site-specific patient characteristics

|  | **Site A** | **Site B** | **Site C** | **Site D** | **Site E** | **Site F** |
| --- | --- | --- | --- | --- | --- | --- |
| **Gender (Male)** |  |  |  |  |  |  |
| Non-AATD | 5985 (61.2%) | 6697 (63.79%) | 2251 (60.35%) | 3959 (59.80%) | --- | 5859 (62.01%) |
| AATD | 91 (62.33%) | 33 (57.89%) | 15 (71.43%) | 55 (64.71%) | --- | 280 (64.52%) |
| **Nicotine** **Abuse** |  |  |  |  |  |  |
| Non-AATD | 2180 (22.29%) | 1512 (14.4%) | --- | 1152 (17.4%) | 146 (6.54%) | 1737 (18.38 %) |
| AATD | 24 (16.44%) | 11 (19.3%) | --- | 29 (34.1%) | 0 (0%) | 106 (24.42 %) |
| **Age at first admission** |  |  |  |  |  |  |
| Non-AATD | 69.37 (13) | 70.65 (12.2) | 70.08 (11.8) | 70.58 (11.72) | 70.44 (11.67) | 70.56 (12.04) |
| AATD | 63.14 (10.76) | 67.05 (13.68) | 65.71 (11.74) | 69.49 (8.33) | 75.00 (9.85) | 70.34 (10.05) |
| **Age at last discharge** |  |  |  |  |  |  |
| Non-AATD | 70.22 (15) | 70.80 (12.21) | 70.12 (11.8) | 70.68 (11.71) | 70.48 (11.66) | 70.65 (12.04) |
| AATD | 64.41 (14.22) | 67.21 (13.64) | 65.71 (11.74) | 69.54 (8.35) | 75.00 (9.85) | 70.51 (10.04) |
| **Number of visits** |  |  |  |  |  |  |
| Non-AATD | 2.34 (2.67) | 2.34 (2.78) | 1.89 (1.84) | 2.12 (2.42) | 1.44 (1.03) | 2.07 (1.96) |
| AATD | 2.91 (3.05) | 3.61 (4.04) | 2.29 (1.87) | 3.27 (3.76) | 2.00 (1.73) | 2.53 (2.46) |
|  |  |  |  |  |  |  |
| **Asthma** |  |  |  |  |  |  |
| Non-AATD | 563 (5.76%) | --- | 25(0.67%) | --- | 10 (0.45%) | 221 (2.34%) |
| AATD | 4 (2.74%) | --- | 0 (0%) | --- | 0 (0%) | 7 (1.61%) |
| **Neutrophils** |  |  |  |  |  |  |
| Non-AATD | 7.45 (5.93) | 10.68 (8.33) | 7.97 (6.09) | 7.59 (6.24) | --- | --- |
| AATD | 8.41 (6.41) | 12.42 (8.55) | 6.66 (5.85) | 7.85 (5.10) | --- | --- |
| **Haemoglobin** |  |  |  |  |  |  |
| Non-AATD | --- | 120.14 (22.76) | 11.81 (2.33) | 11.96 (2.33) | 11.41 (2.33) | 12.76 (2.16) |
| AATD | --- | 112.26 (27.58) | 10.01 (3.16) | 9.96 (1.76) | 10.57 (2.10) | 12.05 (2.25) |
| **Platelets** |  |  |  |  |  |  |
| Non-AATD | 264.44 (126.14) | 262.56 (119.09) | 260.15 (112.92) | 265.24 (123.92) | 262.81 (117.44) | 274.99 (117.41) |
| AATD | 223.66 (126.84) | 250.16 (139.64) | 195.00 (123.80) | 260.46 (147.02) | 313 (90.54) | 255.20 (121.28) |
| **Lymphocytes** |  |  |  |  |  |  |
| Non-AATD | 6.26 (10.33) | 3.65 (7.95) | 1.60 (4.73) | 2.10 (5.52) | --- | --- |
| AATD | 7.98 (11.89) | 2.96 (3.24) | 1.06 (0.47) | 1.18 (0.60) | --- | --- |
| **Procalcitonin** |  |  |  |  |  |  |
| Non-AATD | 2.87 (16.66) | 1.56 (8.07) | 3.00 (9.34) | 1.47 (7.37) | --- | 2.71 (16.11) |
| AATD | 3.13 (10.50) | 0.86 (1.90) | 1.80 (1.61) | 1.69 (7.28) | --- | 0.96 (1.00) |
| **CRP** |  |  |  |  |  |  |
| Non-AATD | 45.29 (64.84) | 45.68 (65.71) | 39.91 (61.11) | 41.73 (58.81) | 36.79 (51.90) | 39.08 (64.56) |
| AATD | 59.33 (71.80) | 41.53 (43.70) | 71.58 (80.20) | 82.15 (77.24) | 29.3 (21.36) | 57.23 (78.52) |
