# Supplementary material for: Alpha-1-antitrypsin-deficiency is associated with lower cardiovascular risk: an approach based on federated learning
Source: Respir Res. 2024 Jan 18;25:38. doi: 10.1186/s12931-023-02607-y (PMC10797985; doi:10.1186/s12931-023-02607-y)
Supplement: Supplementary file 5 — Supplementary Material 5: Table 1: Summary of the variables used in the analysis including the amount of data missing for each laboratory values [file 12931_2023_2607_MOESM5_ESM.docx]

## Supplementary S5

**Table 1:** Summary of the variables used in the analysis including the amount of data missing for each laboratory values.

|  | | **Total**  **N=** **43057**  **Missing (%)** | | **Total non-AATD**  **N=** **42311**  **Missing (%)** | | **Total AATD**  **N=** **746**  **Missing (%)** | |
| --- | --- | --- | --- | --- | --- | --- | --- |
| Creatine Kinase | | 15349 (35.65%) | | 15075 (35.63%) | | 274 (36.73%) | |
| HDL | | 31745 (73.73%) | | 31165 (73.66%) | | 580 (77.75%) | |
| LDL | | 31758 (73.76%) | | 31180 (73.69%) | | 578 (77.48%) | |
| Blood Glucose | | 9613 (22.33%) | | 9437 (22.3%) | | 176 (23.59%) | |
| HbA1c | | 35281 (81.94%) | | 34654 (81.9%) | | 627 (84.05%) | |
| Triglycerides | | 28300 (65.73%) | | 27735 (65.55%) | | 565 (75.74%) | |
| Total Cholesterol | | 28769 (66.82%) | | 28237 (66.74%) | | 532 (71.31%) | |
| ALAT | | 9351 (21.72%) | | 9172 (21.68%) | | 179 (23.99%) | |
| ASAT | | 9723 (22.58%) | | 9561 (22.6%) | | 162 (21.72%) | |
| ^+^ Hs-troponin | | 7683 (63.18%) | | 7603 (63.3%) | | 80 (53.69%) | |

AATD: Alpha 1-Antitrypsin Deficiency, ASAT: Aspartate Aminotransferase, ALAT: Alanine Aminotransferase, HbA1c: Glycosylated haemoglobin, HDL: High Density Lipoprotein, LDL: Low Density Lipoprotein, Hs-Troponin: High sensitivity troponin

^+^ Count for Site A and Site E (Total N= 12161; Total non-AATD = 12012; Total AATD = 149)
