# Supplementary material for: Alpha-1-antitrypsin-deficiency is associated with lower cardiovascular risk: an approach based on federated learning
Source: Respir Res. 2024 Jan 18;25:38. doi: 10.1186/s12931-023-02607-y (PMC10797985; doi:10.1186/s12931-023-02607-y)

**Adjusted influence of cardiovascular comorbidities and respiratory tract infections on COPD**


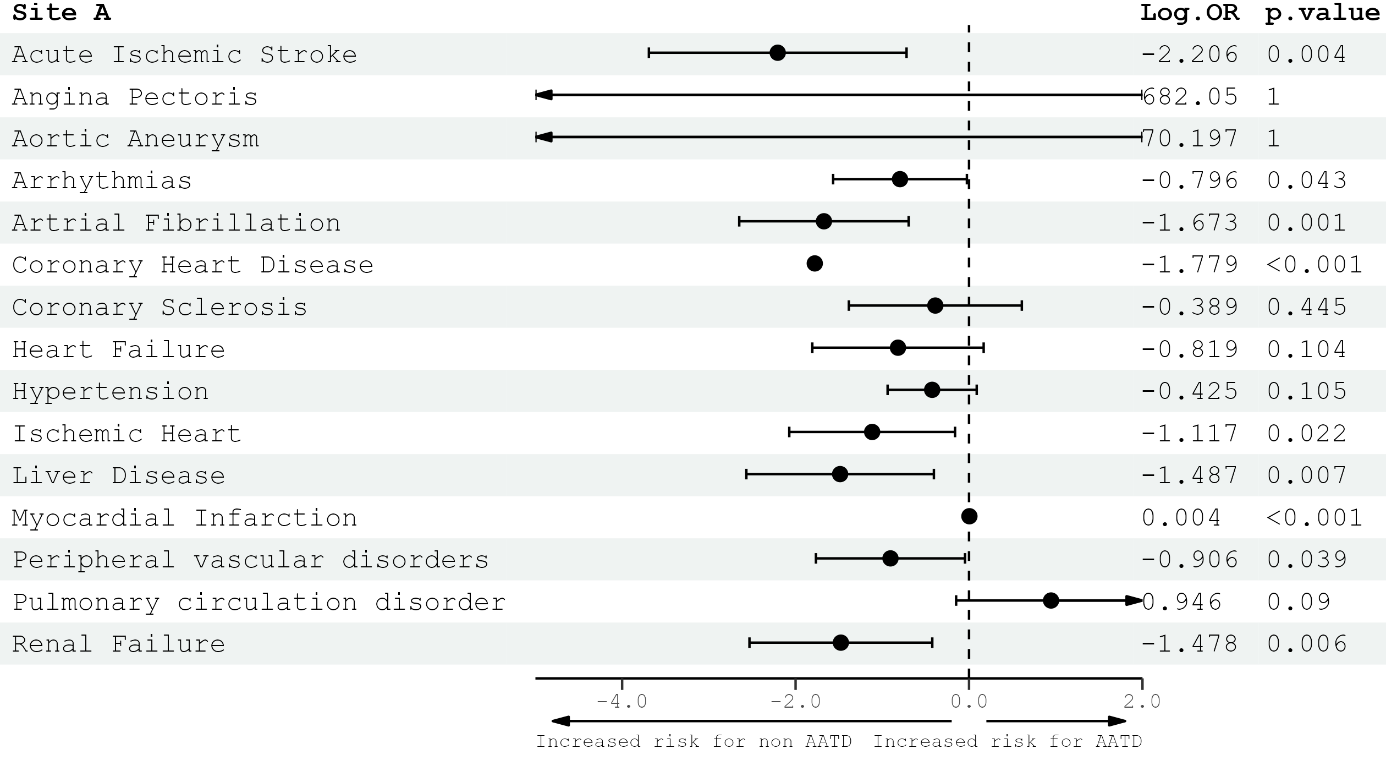


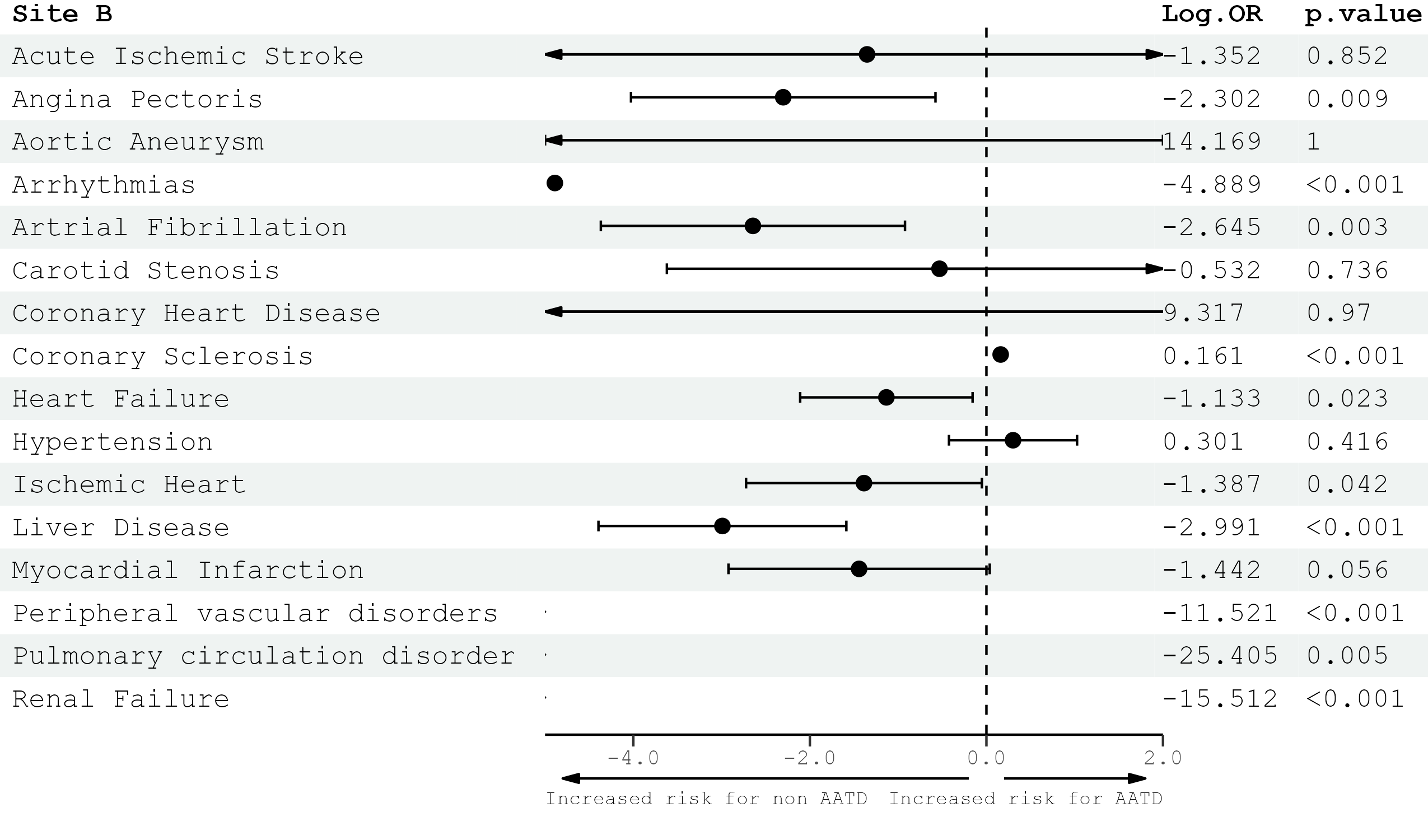

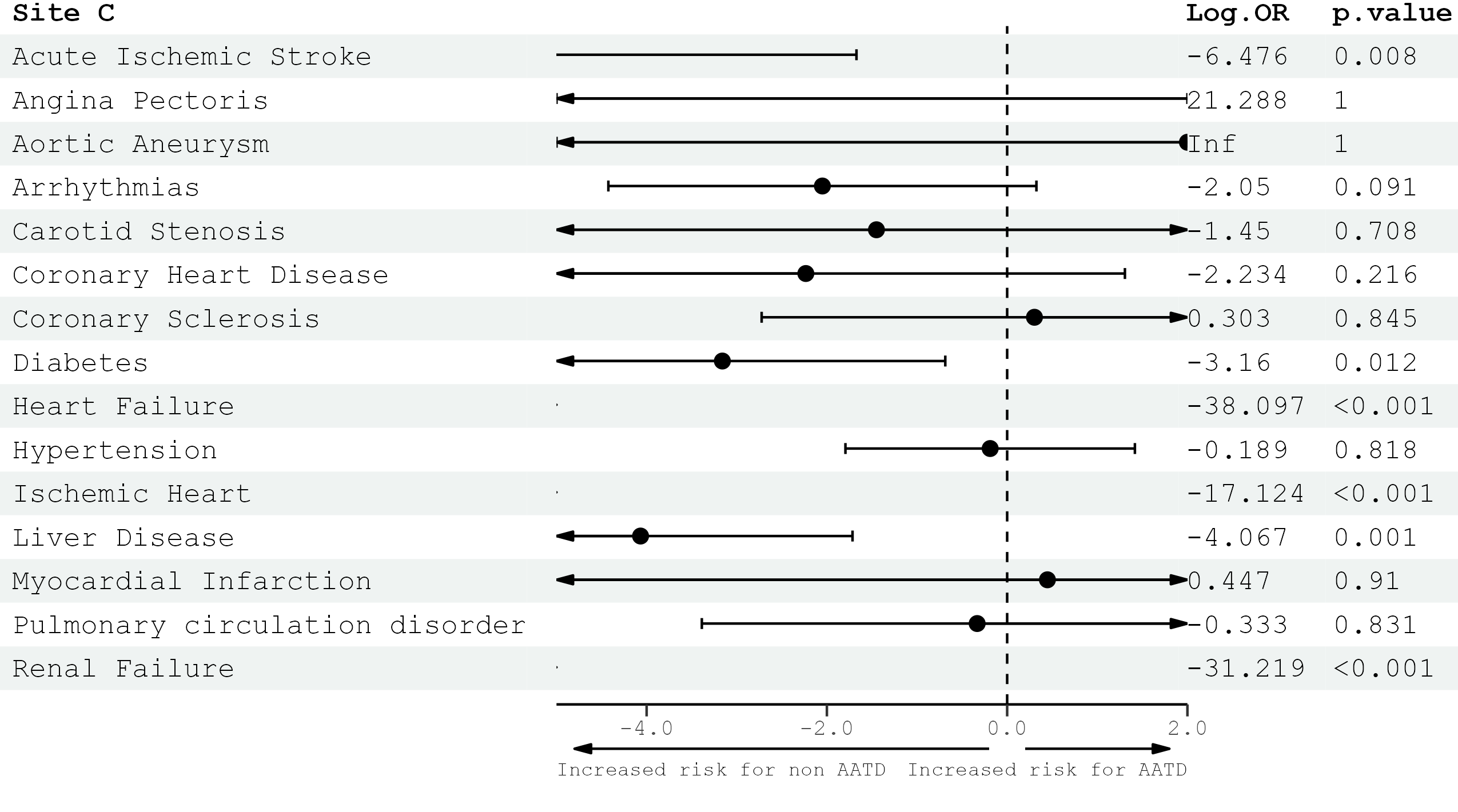


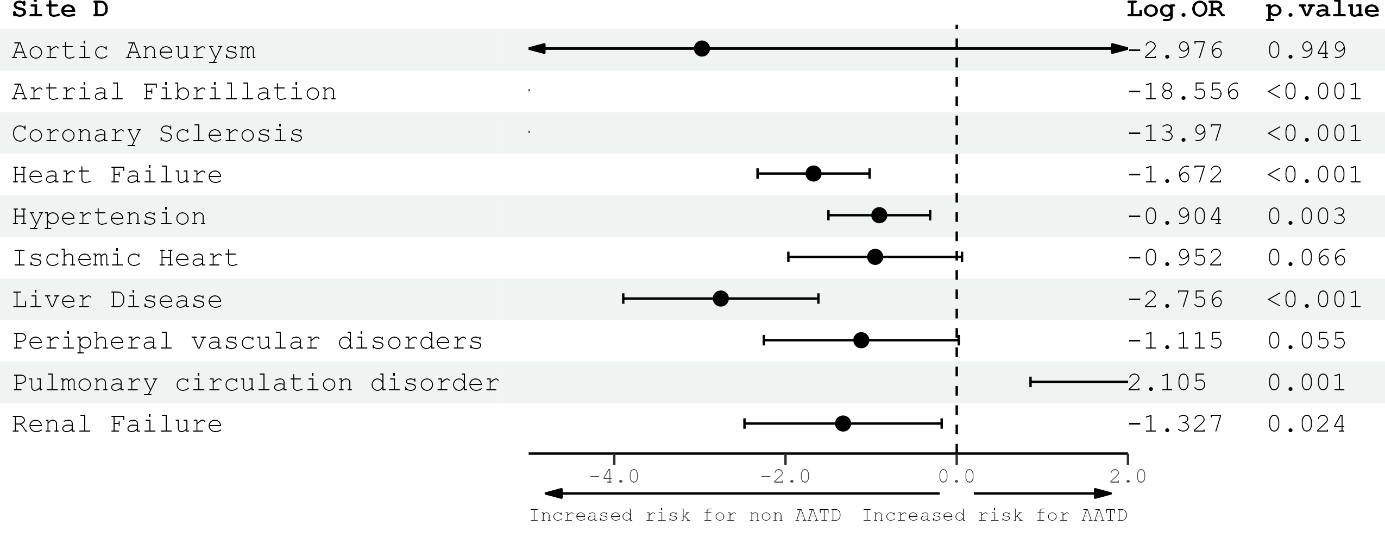


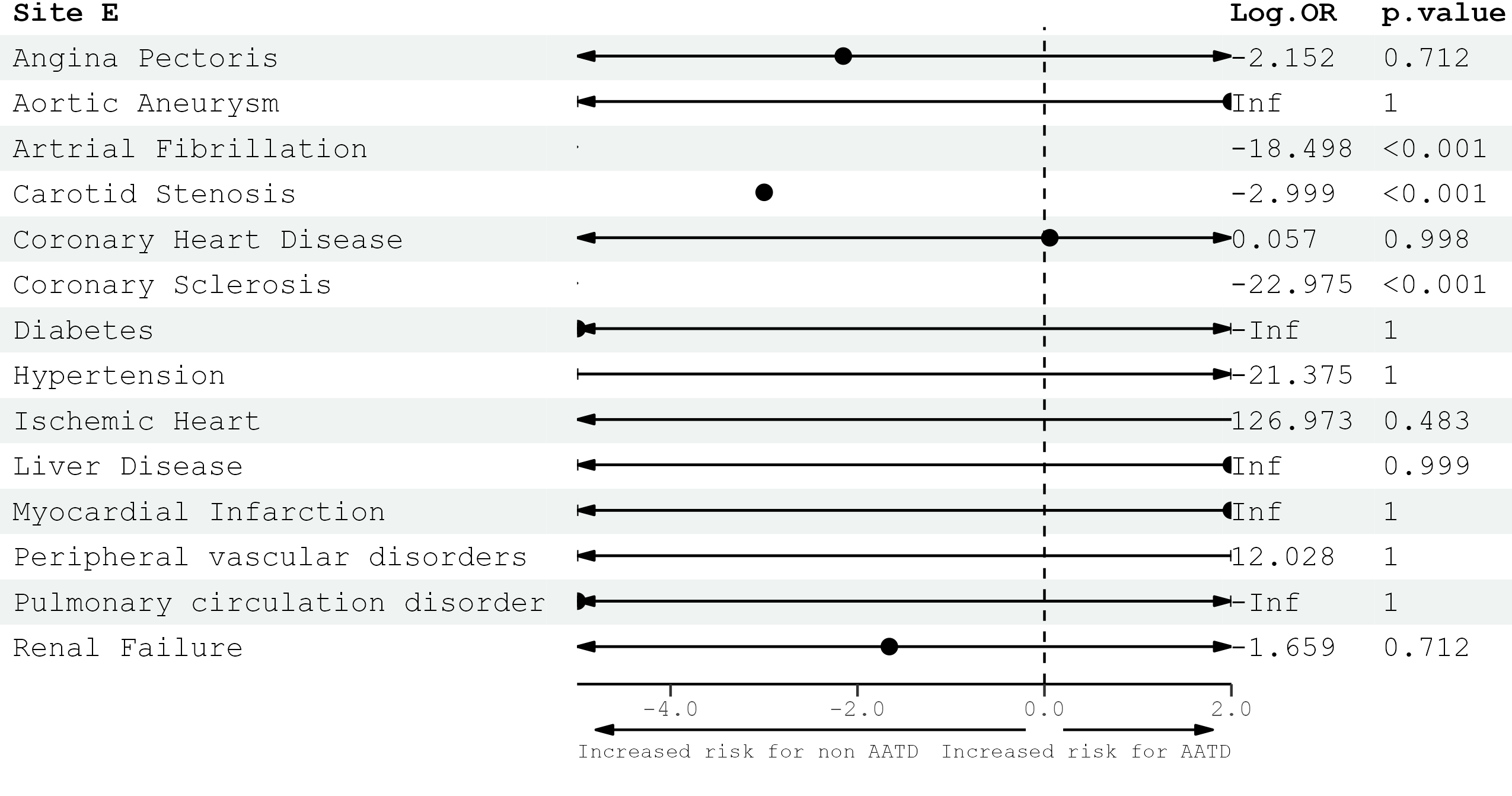


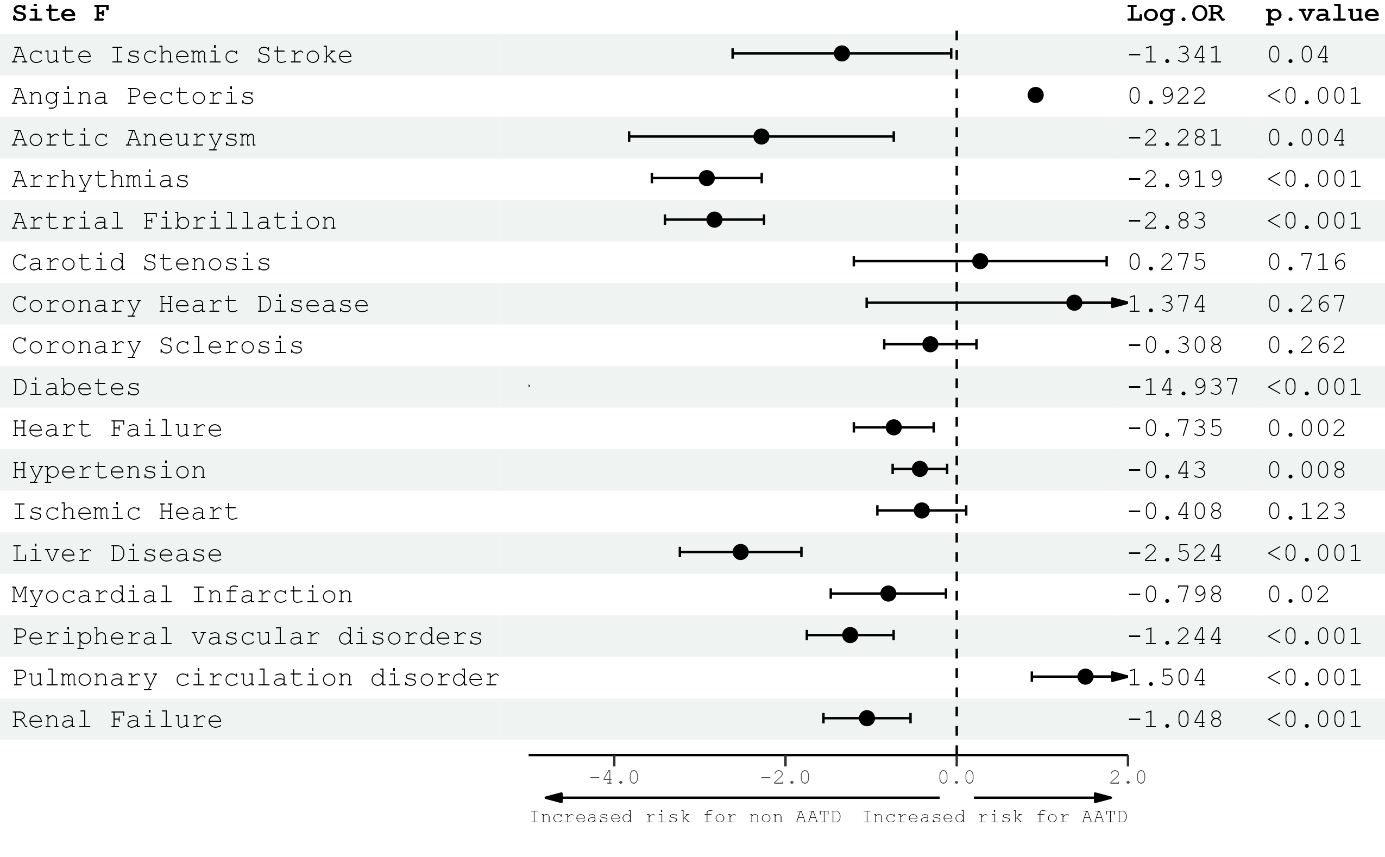

Supplement: Supplementary file 6 — Supplementary Material 6: Site-specific patient characteristics [file 12931_2023_2607_MOESM6_ESM.docx]
